# Novel Putative Effectors Identified in the Arrhythmogenesis of Idiopathic Outflow Tract Ventricular Arrhythmias: A Novel Concept Beyond Triggered Activity

**DOI:** 10.3390/jcm14113957

**Published:** 2025-06-04

**Authors:** Tamas Geczy, Rita B. Gagyi, Attila Nemes, Tamas Szili-Torok

**Affiliations:** 1 Department of Cardiology, Cardiovascular Center Bad Bevensen, 29549 Bad Bevensen, Germany; t.geczy@hgz-bb.de; 2 Cardiac Electrophysiology Division, Cardiology Centre, Department of Internal Medicine, University of Szeged, 6725 Szeged, Hungary; gagyi.rita.beata@med.u-szeged.hu (R.B.G.); nemes.attila@med.u-szeged.hu (A.N.)

**Keywords:** idiopathic ventricular arrhythmia, outflow tract, triggered activity, premature ventricular contraction, arrhythmia mechanism

## Abstract

**Background**: The arrhythmogenic mechanism of idiopathic ventricular arrhythmias (VAs) from the outflow tracts (OTs) and adjacent anatomical structures has been described to be triggered activity. However, it is incompletely understood why this focal mechanism mainly originates from the OTs and what factors could precipitate it. The aim of this study was to further elucidate the arrhythmogenic mechanisms underlying focal ventricular arrhythmias originating from the outflow tracts. **Methods**: Six patients referred for catheter ablation of OT-related PVCs were included in this study. Programmed atrial stimulation at the interatrial septum or within the coronary sinus was performed. Pacing at the AV annuli was capable of evoking OT-PVCs with an ECG morphology identical to clinical PVCs by presumably capturing specific fibers within the network of nodal-type tissue of the AV junctional sleeves. **Results**: Based on the analysis of intracardiac electrograms, the observed PVCs could indeed be elicited as a result of prior atrial stimulation. **Conclusions**: Our findings suggest that unique pathways might exist between specific periannular atrial locations and the OTs, the activation of which could result in triggering PVCs from the presumed “exit site” of these pathways in the OTs. These findings might facilitate the development of a novel ablation strategy, which might also include the mapping of atrial locations, in order to identify and ablate the presumed “entry sites” of these special pathways.

## 1. Introduction

The underlying arrhythmogenic mechanism of idiopathic ventricular arrhythmias (IVAs) originating from the ventricular outflow tracts (OTs) (and adjacent anatomical structures like, e.g., aortic sinuses of Valsalva and aorto-mitral continuity) has generally been accepted to be triggered activity induced by cAMP-mediated delayed afterdepolarization (DAD) [1,2,3,4,5,6]. (Note: besides the above-mentioned structures, a minority of triggered-activity-mediated focal IVAs have also been described to originate from more distant locations like the papillary muscles, the moderator band, and epicardial locations.) However, despite significant research efforts in the field, it still remains elusive why this focal mechanism originates mainly in the ventricular outflow tracts (and the above-mentioned anatomic locations) in structurally normal hearts and what specific factors can be held responsible for initiating arrhythmogenesis. The presumably more complex nature of the arrhythmogenic substrate in OT-related IVA is also supported by observations that describe the relative frequent co-existence of supraventricular tachycardia (mainly AVNRT) with IVA [7,8,9], along with other studies that suggest the involvement of preferential pathways of conduction tissue in arrhythmogenesis (based on observations of discrete prepotentials preceding the ventricular signals within this region) [10,11,12,13]. Moreover, we recently reported on the abolishment of OT-related premature ventricular contractions (PVCs) that occurred in parallel with the successful ablation of accessory pathways at the atrioventricular annuli of both the left and right sides of the heart [14]. In the current case series, we add yet another interesting piece to this puzzle by describing the unique findings of an electrophysiology study of six patients with idiopathic VA. All of these patients were referred to our center for catheter ablation of symptomatic PVCs originating from the ventricular outflow tracts (or adjacent perivalvular structures). We demonstrate here that stimulation at specific atrial locations in the vicinity of the atrioventricular annulae was capable of evoking OT-related PVCs with an electrocardiographical morphology virtually identical to the clinical PVCs. Based on these findings, we hypothesize that specific pathways with preferential conduction might exist between certain atrial locations (e.g., periannular network of nodal-type tissue) and the OT regions, which might play a role in the genesis of OT-related IVA. (Such connections also seem to be possible in the case of perivalvular IVAs, which originate either around the mitral or the tricuspid annulus.)

## 2. Methods

**Figure 1:**
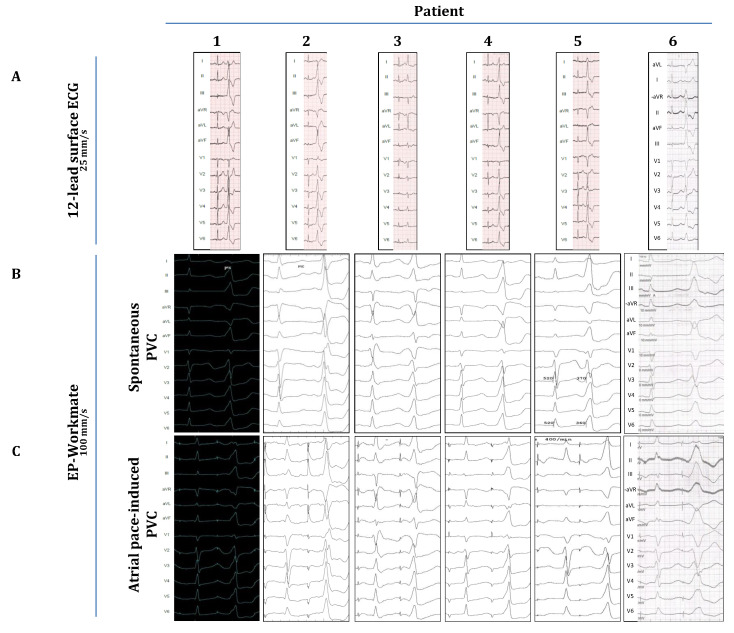
Comparison of spontaneous and atrial-pace-induced PVCs. (**A**) Spontaneous PVCs on pre-procedural ECG (**B**,**C**) spontaneous and atrial-pace-induced PVCs as recorded during procedure with EP-Workmate system.

**Figure 2:**
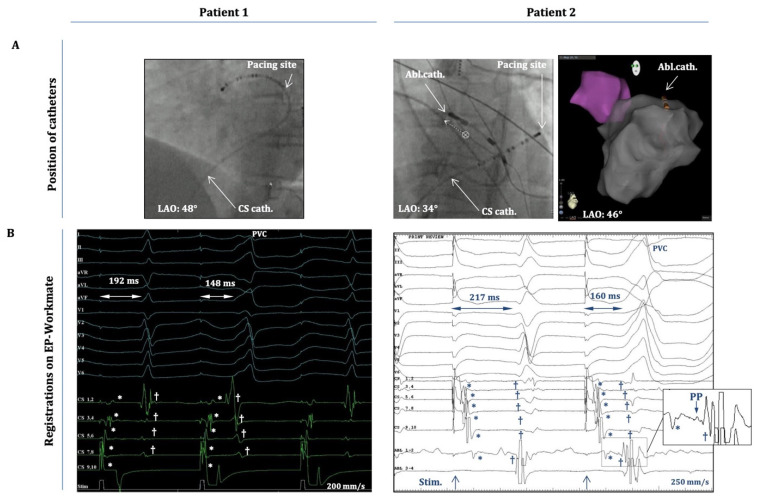
Intracardiac electrograms (EGMs) during atrial-pace-induced PVCs. (**A**) Positions of the decapolar catheter (CS) and the ablation catheter (Abl.) as depicted on fluoroscopy (LAO projection) and on the anatomical map of the CARTO system (in patient 2). Patient 1: The decapolar catheter is located in the distal CS/great cardiac vein, and pacing starts from CS 9,10. Patient 2: The decapolar catheter is located more proximally in the CS (CS 9,10 at CS-ostium), and the ablation catheter is at the superior mitral annulus/aorto-mitral-continuity; pacing starts from CS 1,2. (**B**) Twelve-lead EGMs and intracardiac EGMs from the decapolar/ablation catheters (the latter is only seen for patient 2) as recorded by the EP-Workmate system during an atrial-pace-induced PVC. The timing of the pacing stimulus is depicted at the bottom of each panel. The “atrial-stimulus-to-normal-QRS time” and the “atrial-stimulus-to-PVC time” are depicted on both recordings. Note: The “stimulus-to-PVC time” is shorter in both cases than the “stimulus-to-normal-QRS time”. * Represents the atrial activation signals, and † designates the ventricular activation signals on both panels. Inset) A magnified image of the framed part of the EGM on “Abl1-2” depicting the atrial (*) and ventricular (†) activation signals and a (putative) pre-potential (PP) between them.

Six patients were included in this study. All patients were referred for catheter ablation because of symptoms of palpitations and a significant PVC burden, as detected by 24 h Holter monitoring. A 12-lead surface ECG was performed to assess the origin of the PVCs (Figure 1A). Table 1 contains the demographic data and the findings of echocardiography and cardiac MRI. Table 2 shows the individual PVC burdens together with the morphological analysis of all PVCs on ECG. All patients underwent standard preprocedural preparations: Antiarrhythmic drugs (beta blockers or calcium channel blockers; see Table 1) were discontinued 5 half-lives before the procedure. Local anesthesia (1% lidocaine) was applied to the subinguinal region before vascular access was established. No oral or intravenous sedatives were used during the electrophysiology study in any of the cases described in this study. A radiofrequency ablation catheter (specific type left at the operator’s discretion) was placed into the heart either alone (single-catheter approach) or together with a steerable decapolar catheter (Inquiry, Abbott Laboratories, Abbott Park, IL, USA). The ablation catheter was guided either manually or with magnetic navigation (Stereotaxis MNS; Stereotaxis, Inc., Saint Louis, MO, USA). Despite significant pre-procedural PVC burdens, all patients included in this study exhibited a fairly low PVC frequency (≤1 PVC/3–5 min) during the first phase of the EP procedure, when intravenous isoproterenol (10–20 µg/min) and programmed ventricular stimulation (maximum of three ventricular extra stimuli at two different drive trains from two different locations in the right ventricle using the ablation catheter) were utilized to induce the arrhythmia. Despite these significant efforts, we did not observe OT-related ventricular tachycardia in any of these patients, nor did we detect an increase in their PVC burden. Due to this low PVC frequency, activation mapping was not attempted. Nevertheless, anatomical maps of the OTs were created (Figure 2A, right panel) with the CARTO system (Biosense Webster, Inc., Diamond Bar, CA, USA), and pace-mapping was performed in the OTs to approximate the PVC origins. Subsequently, as part of the attempts to induce clinical PVCs (of OT-origin), programmed atrial stimulation (atrial incremental bipolar pacing at several cycle lengths (CLs) between 350 and 600 ms at an output between 2.5 and 15 mA) was implemented using either the ablation catheter or the decapolar catheter. Atrial stimulation was performed at distinct locations around the mitral annulus (within the coronary sinus, starting at the posteroseptal aspects, and then moving sequentially along the mitral annulus towards the posterolateral, anterolateral, and anteroseptal aspects by advancing the ablation/decapolar catheter further inside the coronary sinus/great cardiac vein) and at the septal aspects of the tricuspid annulus (anteroseptal, midseptal, and posteroseptal). Atrial-pacing-induced PVCs were recorded, and their morphology was compared with spontaneous PVCs (recorded on pre-procedural ECGs and with the EP-Workmate system; Figure 1). The specific periannular sites where OT-related PVCs could be elicited were marked on the fluoroscopic image (Figure 2).

## 3. Results

All patients in this study were referred to our department with symptomatic VA and a high PVC burden (12–19%; ≥10.000 PVC/24 h) despite antiarrhythmic therapy (beta blocker or calcium channel blocker). No patient exhibited clinically relevant valvular disease. Three patients had normal LV systolic function, whereas the other three patients exhibited mild to moderate systolic dysfunction. However, in the latter group, cardiac MRI did not reveal structural heart disease (as evidenced by the lack of late-gadolinium enhancement); hence, all patients were diagnosed to have idiopathic VA, and the latter group was diagnosed to have PVC-induced cardiomyopathy (as the most feasible condition responsible for their LV dysfunction).

Figure 1A demonstrates the clinical PVC of each patient as depicted on the preprocedural ECGs, and Table 2 summarizes the morphological characteristics of these PVCs. In five cases, the clinical PVCs presented with an LBBB morphology, and five clinical PVCs showed a clear inferior axis (except for case 3, where the axis was more horizontal). Based on these characteristics, we concluded that the PVC origins were in the RVOT or the LVOT (depending on transition zones in the precordial leads), except for cases 2 and 3, where they originated from adjacent anatomical structures (case 2: superior mitral annulus/aorto-mitral-continuity; case 3: parahisian region of the RV inflow tract), as also confirmed by pace-mapping during the procedure. Figure 1B shows the relevant PVCs as detected by the EP-Workmate system at a recording speed of 100 mm/s. Figure 1C shows representative images of atrial-pacing-induced PVCs. Their morphology closely resembles that of spontaneous PVCs (Figure 1B,C), i.e., the pacing-induced PVCs show the same type of BBB morphology, the same frontal-plane axis, and identical transition zones.

Importantly, these PVCs occurred after apparent atrial capture; hence, they were not elicited by inadvertent ventricular capture. This is clearly shown in Figure 2, which demonstrates (cases 1 and 2) that the atrial activation sequence on the decapolar electrodes (within the CS) occurs before the onset of the “atrial-pacing-induced” PVC, during pacing starting from CS 9-10 (case 1) or from CS 1-2 (case 2). It is also evident that there is an isoelectric line between the atrial and ventricular local activation signals. In addition, for case 2 (inset in Figure 2B), a small pre-potential between the atrial and ventricular signals on Abl1-2 could also be appreciated (with the ablation catheter located at the spot of earliest ventricular activation). Moreover, the “atrial-stimulus-to-PVC interval” (from the atrial pacing stimulus to PVC onset) was consistently shorter than the “atrial-stimulus-to-normal-QRS interval” (from the pacing stimulus to the onset of the normal QRS) (Figure 2B), and the duration of the “atrial-stimulus-to-PVC interval” (together with local A-to-V intervals on the electrograms from the decapolar/ablation catheters) remained essentially the same when the OT-PVCs were elicited with the same pacing CL during consecutive drivetrains of atrial pacing (the duration of the stimulus-to-PVC intervals did not change by more than 5–10%).

**Figure 3:**
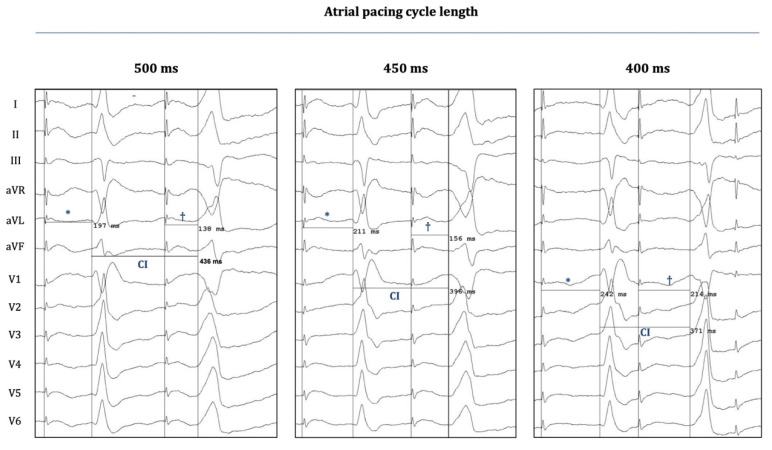
Twelve-lead surface electrograms during atrial-pace-induced PVCs. Surface EGMs (EP-Workmate) are depicted during the occurrence of an atrial-pace-induced PVC (patient 3) at three different atrial pacing cycle lengths (CLs). “Stimulus-to-normal-QRS times” and “stimulus-to-PVC times” are labeled with * and †, respectively; the corresponding durations are indicated on each panel. The coupling intervals (CIs) of PVCs are also indicated. Shortening the pacing CL results in a progressive shortening of the CI, and it also leads to a prolongation of both the “stimulus-to-normal-QRS time” and the “stimulus-to-PVC time”. The latter finding suggests that the putative pathway between the atrium and the OT-locations (entry and exit sites) could possess decremental properties, similarly to AV nodal conduction.

Figure 3 demonstrates another important phenomenon that was observed when OT-PVCs were elicited with progressively shorter atrial pacing CLs: The coupling intervals of the PVCs became progressively shorter when the CL decreased from 500 to 400 ms. On the other hand, decremental conduction was also observed (over the hypothetical preferential pathway between the atrium and the OTs), as the “atrial-stim-to-PVC interval” consistently prolonged with progressively shortened CLs, a phenomenon similar to the characteristic behavior of normal AV conduction over the AV node (which can also be seen in the case of the “normally conducted” atrial pacing stimuli at the left sides of the panels of Figure 3).

**Figure 4:**
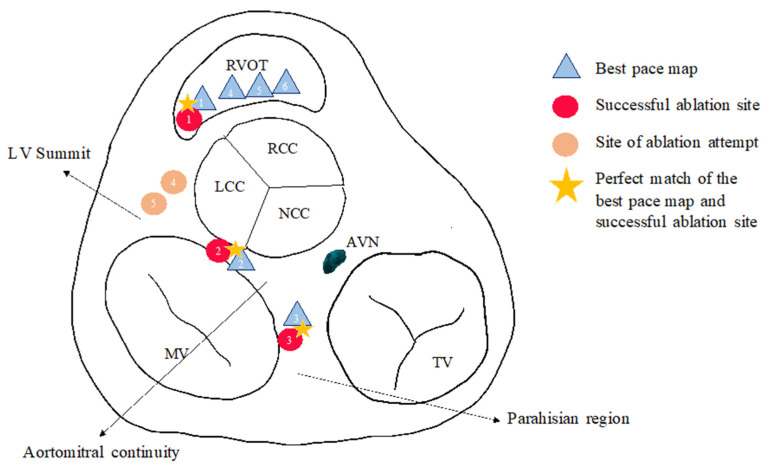
Best mapping vs. ablation site. A schematic presentation of the location of the best pace map (marked with blue triangle) of PVCs and ablation sites (marked with red circle). In some cases, there were only ablation attempts (marked with an orange circle—cases 4 and 5), and in case 6, there was no ablation attempted. The perfect match of the best pace map and successful ablation site are marked with yellow stars. RVOT—right ventricular outflow tract; MV—mitral valve; TV—tricuspid valve; AVN—atrio-ventricular node; RCC—right coronary cusp; LCC—left coronary cusp; NCC—non-coronary cusp.

Figure 4 summarizes the location of the best pace map of the PVCs and successful ablation sites or ablation attempt sites.

Based on these, we concluded that peri-annular atrial pacing was capable to evoke OT-PVCs by presumably capturing the “atrial entry-sites” of certain preferential pathways (between atria and OTs), and due to the decremental behavior of this conduction, it might also be plausible that these unique pathways consist of nodal-type tissue.

## 4. Discussion

Our findings suggest that unique pathways might exist between specific periannular atrial locations and the OTs. The activation of these pathways could potentially trigger PVCs originating from their presumed “exit sites” within the OTs.

### 4.1. Clinical Relevance of OT-VA

Although OT-VAs are generally considered to follow a benign clinical course, there have been reports on (i) clinical cases with more malignant subtypes of OT-VA, capable of causing syncope and even sudden cardiac death, and (ii) incessant forms accounting for tachycardia-induced cardiomyopathy and/or PVC-induced cardiomyopathy [2,3,4]. In addition, high PVC/non-sustained VT burden can render many patients highly symptomatic and therefore significantly lower their quality of life. Thus, according to current guidelines, therapy is recommended in symptomatic cases and in asymptomatic individuals for whom LV dysfunction is suspected to be attributed to PVC-/tachycardia-induced cardiomyopathy [2,3,4,6,15]. In most cases, this means eventual catheter ablation since pharmacological therapy often has limited efficacy [4]. In fact, all of the patients included in this report were referred for catheter ablation due to a high PVC burden with symptoms not amenable to medical therapy, and 50% of these patients were suspected to have LV dysfunction due to PVC-induced cardiomyopathy.

Patients referred to catheter ablation of idiopathic VA represent a significant population in the field of invasive electrophysiology (EP), accounting for around 10% of all referrals to EP centers [16]. The reports on the procedural outcome of OT-VA ablations have been somewhat conflicting with regard to acute success rates and long-term recurrence rates: some studies claimed that the acute and long-term success were nearly 100% [17,18], while others reported more humble outcomes [6,15,19,20]. These varying success rates might reflect differences in follow-up methods and definitions of success and inclusion bias, but it has to be acknowledged that ablation failure in an unselected clinical population is far from being negligible, and there is still room for further improvements. Technical issues, such as (1) the non-inducibility of tachycardia, (2) arrhythmia foci near critical cardiac structures, and (3) epicardial origin, might account for a significant part of ablation failures; however, another important aspect to consider is our limited understanding of the actual arrhythmia substrate underlying IVA.

### 4.2. Arrhythmogenic Substrate for OT-VA: Current Concepts

Delayed afterdepolarization (DAD)-mediated Ca^2+^ overload and subsequent triggered activity (from a focal source in the OT) has generally been accepted to represent the underlying substrate for OT-VA [1,2,3,4,5]. However, the exact etiology of cAMP increase and subsequent Ca^2+^ accumulation is not well understood. Moreover, another unresolved issue is why the OTs would contain the foci of triggered activity in around 80% of cases of IVA [6]. What is so special about them (compared to other areas of ventricles) that would make them more prone to generate triggered-activity-mediated PVCs/VTs?

Abnormal β-adrenergic signaling (increased sympathetic activation or selective dysregulation of β-receptors) within these regions has been implicated to play a crucial role in the pathomechanism [1,5]. Other studies reported subtle structural abnormalities of the RVOT in patients with OTVA, suggesting a possible link to arrhythmogenic right ventricular cardiomyopathy. In contrast, Lermann et al. found no evidence for such structural abnormalities, and they suggest that the unique embryological development of the OT would account for their arrhythmogenic potential. According to this theory, the adult OT is formed by the incorporation of embryonic OT into the working myocardium of the ventricles, which possesses characteristics resembling that of the primary myocardium (slow conduction and spontaneous depolarization); therefore, non-matured remnants of this embryonic phenotype in the adult OT would represent foci with nodal-tissue-like electrical properties, thereby forming the basis for arrhythmogenesis [5,21,22,23].

The RVOT is a primary site for the development of ventricular arrhythmias, such as PVCs, idiopathic ventricular arrhythmias, Brugada syndrome, torsade de pointes, long QT syndrome, and arrhythmogenic right ventricular cardiomyopathy (ARVC) [24]. Its unique developmental origin, cellular composition, and intricate myocardial structure—characterized by high shear wall stress—contribute to its susceptibility to arrhythmias. RVOT myocytes are particularly prone to intracellular sodium and calcium overload, driven by altered calcium handling proteins, increased CaMKII activity, the phosphorylation of ryanodine receptors, and elevated cAMP levels in response to triggering factors or disease states [24].

### 4.3. Conduction Tissue Remnants: Could They Have a Role in Arrhythmogenesis?

Intriguingly, nodal-tissue-like remnants in the OT have also been suggested to derive from the embryologic atrioventricular conduction system. Conduction-tissue-specific immuno-markers have been described to be expressed in the OTs during the early stages of cardiac development [24]. Several reports suggest the possible involvement of remnants of this primitive conduction system in the generation of OT-related IVA based on the observations that discrete pre-potentials occur within the OT (or adjacent structures, e.g., aortic sinuses of Valsalva), which precede the local activation signal when recorded at the spot of the earliest ventricular activation [10,11,12,13]. These reports imply that preferential pathways (insulated from the surrounding myocardium) might exist within these regions, which could connect the origins of the ventricular arrhythmia with their presumable exit/breakout site(s) within the OTs. These studies hypothesize that the anatomical basis for the existence of such insulated pathways (with slow conduction and possible decremental properties) could be conduction tissue that fails to regress during the maturation process of the AV conduction system.

In fact, the entire AV conduction system has been shown to develop from a specialized interventricular ring, and during further steps of the early developmental stages, this primitive conduction tissue would encircle both the junctions of the developing ventricles at the AV orifices (designated as, e.g., the right atrioventricular ring bundle) and the roots of the great arteries (designated as the septal branch, also known as the “dead-end-tract”, and the subaortic root branch). In the later phases of the maturation process, these segments around the AV junctions and the great arteries eventually disappear, giving rise to the mature conduction system, which only consists of the AV node, the His bundle, and the bundle branches [25]. However, in case any part of the above-mentioned structures fails to regress completely, this remnant conduction tissue could represent an arrhythmogenic focus at any of the above locations (including the OTs).

Other studies even describe sleeves of nodal-type tissue around the tricuspid and the mitral annuli in the normal adult heart, which are also believed to represent remnants of the primitive conduction system [26]. This AV junctional network of nodal-type tissue exhibits histological characteristics similar to those of atrial tissue, but their cellular electrophysiology rather resembles the characteristics of nodal tissue (action potential and conduction properties, adenosine response, relative lack of gap junctional protein connexin-43). Although their actual function remains rather elusive, studies have shown that certain parts of these AV junctional sleeves can be electrically dissociated from the surrounding atrial tissue; therefore, they might be insulated from the atrial myocardium. Moreover, the posterior approaches of this system towards the AV node have been implicated to be the actual substrate of the slow AV nodal pathway, which represents the essential component of the re-entry circle in AVNRT [26]. Some studies report on the relatively frequent co-existence of AVNRT and OT-Vas [7,8,9]. Intriguingly, Kautzner et al. even describe cases where AVNRT was observed to initiate runs of OT-VT spontaneously and repeatedly. Moreover, in one case, the termination of the RVOT-VT during RF application led to an abrupt transition into typical AVNRT [8].

Boukens et al. report that Wnt plays a key role as a transcriptional regulator in guiding the embryonic development of the atrioventricular canal [27]. Abnormal activation of Wnt signaling in the mouse ventricle can trigger the development of ectopic tissue resembling the atrioventricular junction, characterized by the absence of Cx40 and Scn5a expression and by slow electrical conduction. Mutations in plakoglobin, which disrupt Wnt signaling, have been linked to ARVC [27].

### 4.4. PVC Ablation from the Atrium?

We recently reported on the unique electrophysiological findings of patients with co-existent WPW syndrome and OT-PVCs. The successful ablation of accessory pathways (APs) at the atrioventricular annuli simultaneously abolished the occurrence of the concomitant PVCs originating from the OTs [14]. These observations suggest that during the ablation of the Aps, we might have also ablated parts of the above-mentioned network of nodal-type tissue within the AV junctional sleeves, thereby involuntarily disrupting the possible “entry” sites of those preferential pathways (of conduction tissue remnants) that have been suggested to have exit sites in the OTs. A similar explanation might also account for the unique results that Garcia et al. present in their report, which describes (for the first time in the literature) the approach of idiopathic VA ablation from the right atrium. The authors suggest that the feasibility of targeting specific PVCs (arising from the posterior–superior process of the left ventricle) from the right atrium lies in the fact that these structures are in close proximity to each other [28]. However, an alternative explanation for these unique findings could be the ablation of the “entry” sites of the above-mentioned specific pathways in the AV junctional network at the tricuspid annulus.

### 4.5. Atrial Pacing-Induced OT-PVCs: Implications on Novel Mechanistic Insights into Arrhythmogenesis

Following the logic of this hypothesis in the current report, we show that atrial pacing around the AV annuli was capable of evoking OT-PVCs (Figure 1, Figure 2 and Figure 3) by presumably capturing specific fibers within the network of the nodal-type tissue of the AV junctional sleeves. Although these OT-PVCs did not follow each atrial pacing stimulus, they occurred relatively frequently during consecutive drivetrains of pacing at certain locations in the periannular regions. Based on our recordings, it was evident that the PVCs did not occur due to an inadvertent ventricular capture, but instead, after apparent atrial activation (Figure 1 and Figure 2). Although the possibility for the concomitant occurrence of *spontaneous* PVCs during pacing episodes may not be excluded unambiguously, the following characteristics might argue for a causative interrelation between atrial pacing and the induction of OT-PVCs: (1) The selected patients exhibited a fairly low frequency (≤1 PVC/3–5 min) of spontaneous PVCs throughout the whole course of the EP study (as mentioned above in the Methods (Section 2)), whereas with atrial pacing, we could reproducibly induce PVCs (and hence significantly enhance their frequency of occurrence). (2) The duration of the “atrial-stimulus-to-PVC interval” remained relatively constant when pacing with the same cycle length. (3) Subtle morphological differences could indeed be detected between spontaneous and pace-induced PVCs (Figure 2B,C). (4) The coupling intervals of the pace-induced PVC (from the preceding normal QRS) showed a progressive shortening with decreasing pacing cycle lengths, whereas we observed a fairly constant coupling interval (at a certain range of heart rate) in the case of spontaneous OT-PVCs. Taken together, all of these findings suggest that the observed PVCs could indeed be elicited as a result of prior atrial stimulation and that the co-incidental occurrence of spontaneous PVCs during atrial pacing might be excluded (although the latter scenario cannot be completely excluded either).

We therefore speculate that unique connections (conduction tissue remnants?) might exist between specific atrial locations and the OTs, the activation of which could result in triggering PVCs (or repetitive ventricular activation) from the presumed exit point of these pathways in the OTs. In the presence of insulation defects, such special “atrium-to-outflow-tract” pathways could represent the pathophysiological substrate of OT-VA. Since this premature conduction tissue network has previously been indicated (i) to be partially insulated from the surrounding (atrial) myocardium and (ii) to possess distinct conduction properties, it is plausible to assume that they could serve as preferential pathways between the outflow tract and the atria, and once excited (either spontaneously or by pacing), their activation wave-front might be able to occasionally “leak out” towards the outflow tract regions and exit there in the form of an OT-PVC. (The above-mentioned subtle morphological differences between the spontaneous and pace-induced PVCs might be explained by the existence of multiple adjacent exit points for the network of these conduction fibers, located within close proximity to each other within the same region of the OTs, and activated somewhat differently during pacing than during spontaneous PVCs.) That said, this remains speculative, and other mechanisms cannot be ruled out; therefore, further research is necessary to clarify the underlying processes.

### 4.6. The Limitations of the Study

One key limitation of this study is the small sample size of only six patients, which limits the statistical power to generalize the findings or establish causality. However, we also note that multiple case series of similar scale have historically played an important role in facilitating future research and uncovering critical mechanisms, suggesting that preliminary insights from small cohorts can still be valuable in guiding subsequent studies.

Another limitation of this study is the lack of comparison with non-OT-VA patients or sham pacing protocols, which may weaken the specificity of our findings. Including such comparisons could help determine whether the observed effects are unique to OT-VA substrates. While this was beyond the scope of our current case series, we acknowledge the importance of this approach and anticipate that future prospective, large-scale studies—ideally conducted by independent groups—will help validate and extend our findings, potentially even in patients without PVCs.

Another important limitation of this study is that we cannot completely exclude the possibility that atrial pacing had a non-specific effect on the induction of PVCs. Such non-specific effect could be the generally increased sympathetic tone, which is caused by the rapid heart rate during fast atrial pacing, which feels unusual and uncomfortable for the patient. This could then lead to increased beta-adrenergic signaling, which would in turn increase the possibility of triggered activity. However, in such a scenario, it would still be controversial why PVCs would only originate from the outflow tracts and not from any other part of the heart. Nevertheless, we would like to emphasize that further research is required in order to find compelling evidence for the existence of these unique atrial-to-outflow-tract pathways and their potential role in the pathogenesis of IVAs. Furthermore, we must emphasize that although our findings obtained from this series of cases raise concerns about generally accepted mechanisms in the development of idiopathic PVC, it is not possible to exclude the role of sympathetic activation (due to pacing-induced tachycardia) in non-specifically triggering PVCs.

By describing the intriguing phenomenon of atrial-pace-induced OT-PVCs in our patients, we wished to add an important “puzzle piece” to these research efforts. We also believe that the importance of our present report lies in the fact that (together with previous observations) it could have the potential to transform our current approach of performing catheter ablation of OT-VA, since it might introduce a novel strategy for extending our mapping efforts into the atria, in order to identify and ablate the “entry sites” of these special “atrium-to-outflow-tract” pathways.

## Figures and Tables

**Table 1:** Patient characteristics and echocardiographic/cardiac MRI findings.

	Age	Sex (m/f)	LVEF (%)	Structural Heart Disease	Antiarrhythmic Medication
**Case 1**	52	m	45 †	None *	Beta blocker
**Case 2**	57	f	45 †	None *	Beta blocker
**Case 3**	58	f	60	None	Calcium channel blocker
**Case 4**	57	f	64	None	Calcium channel blocker
**Case 5**	71	m	36 †	None *	Beta blocker
**Case 6**	43	f	67	None	Beta blocker

LVEF = left ventricular ejection fraction; * As revealed by cardiac magnetic resonance imaging. † Improvement in LVEF during follow-up.

**Table 2:** ECG/Holter findings.

	PVC Burden		PVC Morphology			Possible PVC Origin
	Percentage	>10.000 /24 h	BBB Pattern	Horizontal Axis	Precordial Transition Zone	
**Case 1**	17%	yes	LBBB	Left inferior	V2–V3	outflow tract
**Case 2**	12%	yes	RBBB	Right inferior	none	aortomitral continuity/superior mitral annulus
**Case 3**	16%	yes	LBBB	Horizontal	V3–V4	parahisian region
**Case 4**	16%	yes	LBBB	Left inferior	V1–V2	outflow tract
**Case 5**	19%	yes	LBBB	Left inferior	V2–V3	outflow tract
**Case 6**	18%	yes	LBBB	Inferior	V2–V3	outflow tract

PVC = premature ventricular contraction; BBB = bundle branch block; LBBB = left bundle branch block; RBBB = right bundle branch block.

## Data Availability

The data that support the findings of this study are available from the corresponding author (T.S.-T.) upon reasonable request.

## Abbreviations

AVNRT	atrioventricular nodal reentrant tachycardia
CS	coronary sinus
cAMP	cyclic adenosine monophosphate
DAD	delayed afterdepolarization
IVA	idiopathic ventricular arrhythmias
ECG	Electrocardiogram
L/RVOT	left/right ventricular outflow tract
OT	outflow tract
LV	left ventricle
LBBB	left bundle branch block
RBBB	right bundle branch block
BBB	bundle branch block
PVC	premature ventricular contraction
EP	electrophysiology
AP	accessory pathway
VA/T	ventricular arrhythmia/tachycardia
